# Survival prediction in Amyotrophic lateral sclerosis based on MRI measures and clinical characteristics

**DOI:** 10.1186/s12883-017-0854-x

**Published:** 2017-04-17

**Authors:** Christina Schuster, Orla Hardiman, Peter Bede

**Affiliations:** Quantitative Neuroimaging Group, Academic Unit of Neurology, Room 5.43, Biomedical Sciences Institute, Trinity College Dublin, Pearse Street, Dublin 2, Ireland

**Keywords:** Amyotrophic lateral sclerosis, Magnetic resonance imaging, Biomarker, Diffusion tensor imaging, Cortical thickness, Binary logistic ridge regression, Cross-validation, Independent validation, Prognosis

## Abstract

**Background:**

Amyotrophic lateral sclerosis (ALS) a highly heterogeneous neurodegenerative condition. Accurate diagnostic, monitoring and prognostic biomarkers are urgently needed both for individualised patient care and clinical trials. A multimodal magnetic resonance imaging study is presented, where MRI measures of ALS-associated brain regions are utilised to predict 18-month survival.

**Methods:**

A total of 60 ALS patients and 69 healthy controls were included in this study. 20% of the patient sample was utilised as an independent validation sample. Surface-based morphometry and diffusion tensor white matter parameters were used to identify anatomical patterns of neurodegeneration in 80% of the patient sample compared to healthy controls. Binary logistic ridge regressions were carried out to predict 18-month survival based on clinical measures alone, MRI features, and a combination of clinical and MRI data. Clinical indices included age at symptoms onset, site of disease onset, diagnostic delay from first symptom to diagnosis, and physical disability (ALSFRS-r). MRI features included the average cortical thickness of the precentral and paracentral gyri, the average fractional anisotropy, radial-, medial-, and axial diffusivity of the superior and inferior corona radiata, internal capsule, cerebral peduncles and the genu, body and splenium of the corpus callosum.

**Results:**

Clinical data alone had a survival prediction accuracy of 66.67%, with 62.50% sensitivity and 70.84% specificity. MRI data alone resulted in a prediction accuracy of 77.08%, with 79.16% sensitivity and 75% specificity. The combination of clinical and MRI measures led to a survival prediction accuracy of 79.17%, with 75% sensitivity and 83.34% specificity.

**Conclusion:**

Quantitative MRI measures of ALS-specific brain regions enhance survival prediction in ALS and should be incorporated in future clinical trial designs.

**Electronic supplementary material:**

The online version of this article (doi:10.1186/s12883-017-0854-x) contains supplementary material, which is available to authorized users.

## Background

Amyotrophic lateral sclerosis is a relentlessly progressive neurodegenerative condition. While the clinical features of ALS are highly heterogeneous, the overall disease trajectory and life expectancy from diagnosis is relatively uniform, making it a template neurodegenerative condition for the development of diagnostic and prognostic biomarkers [1]. It is widely accepted that a long pre-symptomatic phase precedes the clinical manifestation of ALS [2] which may be dominated by bulbar or spinal symptoms at onset, but progresses to respiratory failure over time.

Clinical heterogeneity has multiple dimensions in ALS such as site of onset, coexisting cognitive and behavioural deficits, dominance of upper or lower motor neurodegeneration, variability in progression rates and the relatively distinct clinical profile of various ALS genotypes [3, 4]. All of these variables make accurate individual prognostication particularly challenging. Clinical heterogeneity precludes smaller clinical trials [5] as a given drug may only be effective in certain ALS phenotypes. Robust and validated prognostic frameworks would enhance patient stratification into clinical trials, and enable the optimised management of individual patients. The planning and timing of supportive interventions such as feeding tube insertion, initiation of non-invasive ventilation, addressing end-of-life decisions and palliative measures could be guided by accurate prognostic markers.

Previous studies of ALS have successfully linked specific demographic and clinical variables to shorter survival; e.g. older age, bulbar or respiratory onset, recent symptom onset prior to diagnosis, significant motor impairment, coexisting executive dysfunction, rapid weight loss [6–9]. Attendance of a multidisciplinary ALS clinic has been linked to a better prognosis [10].

Magnetic resonance imaging (MRI) has been repeatedly proposed as a diagnostic or prognostic biomarker in ALS. [11, 12] The core imaging features of ALS related neurodegeneration are well described: degeneration of the precentral gyrus [13, 14], corpus callosum and corticospinal tract [15, 16]. Despite numerous descriptive imaging studies in ALS, few studies have successfully translated group-level findings to aid the interpretation of individual data sets. While imaging measures were repeatedly proposed as potential diagnostic biomarkers in ALS, imaging parameters of single anatomical structures led to relatively poor diagnostic classification accuracy [17–20]. Imaging measures in ALS have also been explored as prognostic indicators. Neuronal integrity of the motor cortex has been directly linked to survival [21] and corticospinal tract fractional anisotropy (FA) was used to predict 3-year survival [22].

The objective of this study is to develop and test an objective prognostic tool in ALS to predict 18-month survival based on quantitative MRI data. We hypothesised that structural MRI measures enhance prediction accuracy compared to clinical variables alone.

## Methods

### Methods overview

**Fig. 1:**
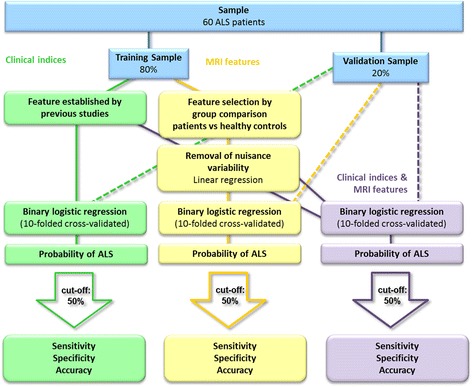
Overview of methods

Patients were divided into a “training sample” and an “independent validation sample”, to develop and test a binary logistic ridge regression model predicting 18-month survival **(**Fig. 1). Core clinical parameters relating to survival were selected based on a comprehensive literature review. ALS-specific, MRI measures were selected based on group comparisons between the patients of the training cohort and healthy controls. Survival prediction accuracy was evaluated based on (**a**) clinical indices alone, (**b**) MRI measures alone and (**c**) based on both clinical and MRI measures.

### Participants

Data were acquired prospectively as part of the biomarker initiative of the Academic Unit of Neurology in Trinity College. Written informed consent was provided by every participant in accordance to the Medical Ethics approval of the research project (Ethics (Medical Research) Committee - Beaumont Hospital, Dublin, Ireland). Inclusion criteria included a diagnosis of definite or probable ALS according to the revised El Escorial criteria [23] and the ability to lie supine in the MRI scanner for 45 min. Exclusion criteria included unexpected radiological findings, coexisting psychiatric conditions, previous traumatic head injury, poorly controlled diabetes, hydrocephalus, prior haemorrhagic or ischaemic stroke. Patients with comorbid frontotemporal dementia according to the Rascovsky Criteria were also excluded because of the confounding imaging changes associated with this phenotype [24].

Based on the above criteria 69 consecutive ALS patients were prospectively enrolled and scanned between January 2011 and January 2015. Out of the 69 patients 3 patients could not tolerate the duration of the MRI protocol, and unexpected imaging findings were identified in 3 patients; falcine meningioma, gliotic cortical changes secondary to an old head injury, and a small old parietal stroke. Survival data was reviewed based on the national ALS-register in June 2016. Out of the 63 patients included, 33 were alive more than 18 months after their scan and 30 patients passed away within 18 months. To provide equally sized study groups of short and long survivors, 30 patients were randomly selected out of the 33 patients who were alive 18 months after their scans. 80% of the patients were randomly allocated to the training sample and 20% to the validation sample. In order to highlight the ALS specific pathology, the training sample was compared to an age- and gender matched group of 69 healthy controls. [25] The demographic and clinical profile of the participants in presented in Table 1.

**Table 1:** Clinical and demographic data of study participants

			Training Sample			Validation Sample		
	Healthy controls		ALS patients surviving			ALS patients surviving		
		*p*-value*	< 18 months	> 18 months	*p*-value	< 18 months	> 18 months	*p*-value
n	69		24	24		6	6	
Gender (male/ female)	34/35	*P* = 0.22	17/7	13/11	*P* = .37	3/3	2/4	*P* = 1
Handedness (right/left)	64/5	*P* = .55	23/1	20/4	*P* = .35	5/1	5/1	*P* = 1
Age, years (means, SD**)	59.97 (9.9)	*P* = .17	63.18 (8)	61.76 (10.73)	*P* = .60	63.96 (8.03)	55.09 (8.82)	*P* = .09
Site of onset (bulbar/spinal)			8/16	10/14	*P* = .76	3/3	2/4	*P* = 1
Diagnostic delay, years (mean, SD)			1.2 (0.81)	1.05 (0.75)	*P* = .51	1.28 (1.1)	0.88 (0.3)	*P* = .43
Disease duration from symptom onset until scan, years (mean, SD)			2.17 (1.01)	2.32 (1.34)	*P* = .67	1.94 (1.44)	1.85 (0.54)	*P* = .89
ALSFRS-r (mean, SD)			34.48 (6.84)	37.38 (6.21)	*P* = .12	34.5 (8.34)	39.17 (4.17)	*P* = .25
King’s College Staging (1/ 2/ 3/ 4/ unavailable)			4/5/3/7/5	4/9/5/4/2		0/1/2/2/1	2/2/1/1/0	
MITOS Staging (0 /1/ 2/ 3/ unavailable)			10/8/1/0/5	19/2/0/1/2		3/1/1/0/1	6/0/0/0/0	
Survival from scan, years (mean, SD)			0.94 (0.32)	2.26 (1.11)	*P* < .01	0.92 (0.26)	2.62 (1.32)**	*P* < .05

*healthy controls were compared to the training sample of 48 patients. **SD standard deviation

### Imaging data acquisition

MR data were acquired on a 3 Tesla Philips Achieva MRI platform with a maximum gradient strength of 80mT/m using an 8-channel receive-only head coil. T1-weighted images were obtained using a three-dimensional inversion recovery prepared spoiled gradient recalled echo (IR-SPGR) sequence with a field of new (FOV) of 256 × 256 × 160 mm, spatial resolution = 1 mm3, TR/TE = 8.5/3.9 ms,TI = 1060 ms, flip angle = 8°, SENSE factor = 1.5. Diffusion Tensor Images (DTI) were acquired using a spin-echo planar imaging (SE-EPI) sequence with a 32-direction Stejskal-Tanner diffusion encoding scheme: FOV = 245 × 245 × 150 mm, spatial resolution = .5 mm3, 60 slices with no interslice gap, TR/TE = 7639 / 59 ms, SENSE factor = 2.5, b-values =0, 1100 s/mm2, with SPIR fat suppression and dynamic stabilisation in an acquisition time of 5 min 41 s.

### Imaging data analyses

#### MRI pre-processing

##### White matter (WM) analysis

The pre-processing of the diffusion weighted images included eddy current corrections, motion corrections, and brain-tissue extraction in FSL [26]. A diffusion tensor model was fitted at each voxel, generating maps of fractional anisotropy (FA), mean diffusivity (MD), axial diffusivity (AD), and radial diffusivity (RD). Each dataset was aligned to the FMRIB58a_FA standard-space images. Next, a mean FA image was created. Each subject’s aligned FA data was then projected onto the FMRIB58a_FA standard-space skeleton and the resulting data fed into voxel-wise cross-subject statistics.

##### Cortical thickness (CT) analysis

Cortical thickness was evaluated using the FreeSurfer imaging analysis suite (http://surfer.nmr.mgh.harvard.edu/; version 5.3.0), which has been both validated using histological [27] and manual measurements [28]. The automated processing pipeline consists of skull-stripping, registration, intensity normalization, Talairach transformation, tissue segmentation, and surface parcellation. Tissue segmentation determines the white/grey matter interface (white matter surface) and grey matter/cerebrospinal boundary (pial surface). The result of this process was individually reviewed, and if errors were detected the segmentation step was repeated. Cortical thickness has been defined as the distance (vertex) from the white matter surface to the nearest point on the pial surface.

#### Feature selection

**Fig. 2:**
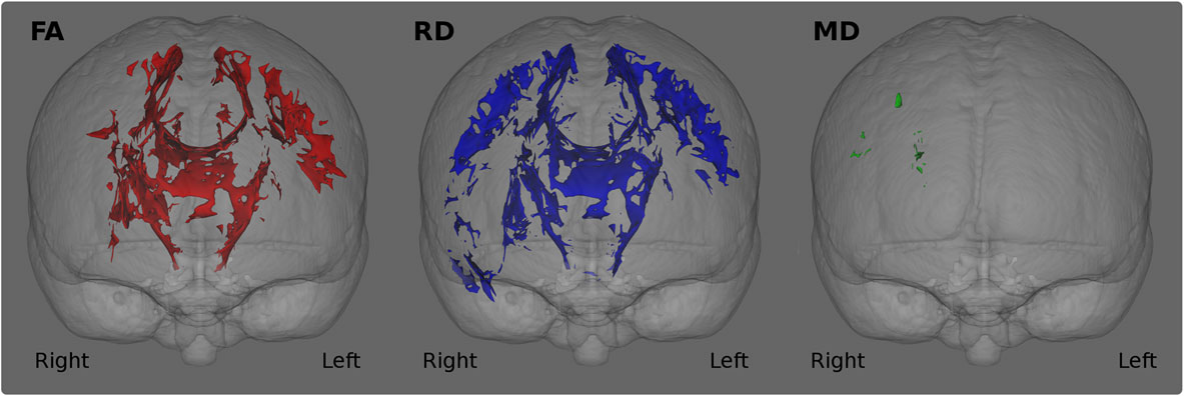
White matter analyses. Group comparisons between ALS patients and controls. The significance level is set to *p* < .01 FWE

**Fig. 3:**
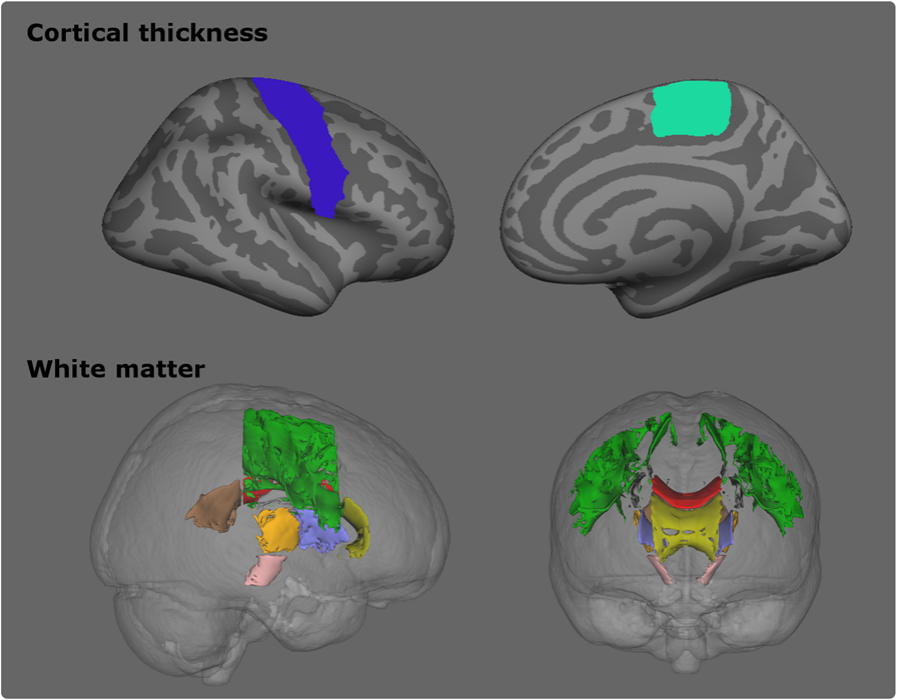
MRI features included in the regression analyses. *Top*: Cortical thickness. *Blue*: precentral gyrus, *Light green*: paracentral gyrus. Only the *right* hemisphere is displayed. *Bottom*: *White* matter. *Green*: superior corona radiata, *grey*: corona radiata, *orange*: posterior limb of the internal capsule, *lilac*: anterior limb of the internal capsule, *rose*: cerebral peduncle, *yellow*: genu of corpus callosum, *red*: body of the corpus callosum, *brown*: splenium of the corpus callosum

In order to identify ALS-specific pathological brain regions, patients of the training sample were compared to healthy controls using age as a nuisance variable. The significance level for the group comparisons were set to *p* < 0.01 corrected using family-wise error (FWE). Figure 2. Based on these analyses, the following core white matter regions were selected as discriminatory features for the binary ridge regression: the superior corona radiata, inferior corona radiata, anterior and posterior limbs of the internal capsule, cerebral peduncles (mesencephalic crus) and the genu, body and splenium of the corpus callosum. Figure 3. These regions were defined based on the above patients-versus-controls contrast using the JHU DTI-based white-matter atlas labels. The average value of each DTI index (i.e. FA, RD, MD, AD) was extracted from each white matter region.

**Fig. 4:**
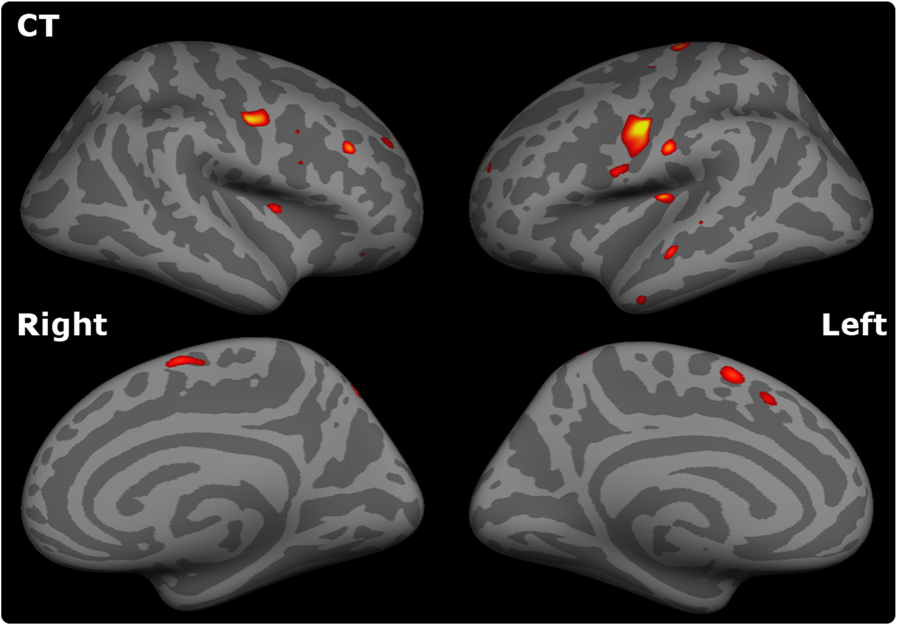
Cortical thickness analyses. Group comparison between ALS patients and controls. The significance level is set to *p* < .05 FDR

The comparative cortical thickness analyses were significant at *p* < .05 corrected for multiple comparisons using false discovery rate (FDR). Figure 4. Cortical features were selected based on these comparisons and expanded to include the entire precentral gyrus and the paracentral gyrus based on available literature [29, 30]. The Desikan-Killiany atlas [31] was utilised to define the cortical regions and the average cortical thickness was extracted from both regions.

For all variables, values in left and right hemisphere were averaged. The pre-processing steps of the independent validation sample were analogous to the pre-processing pipeline of the training sample.

#### Reducing age-related variability

The effect of aging on MRI measures is well established and it has been shown that classification accuracy may be improved by removing this nuisance variable. The confounding effect of age is particularly important in ALS which affects a fairly a wide age range. From an imaging perspective, a young patient with severe physical disability may exhibition similar brain changes to older patients with less advanced disease. Moreover, age in ALS is considered a prognostic factor. [8, 32] To account for age-related variability the method of Koikkalainen et al. was implemented [33]. A linear regression model was fitted to the distribution of the values of each feature of the control group using age as independent variable. Based on this equation, we estimated the predicted value for each feature for each individual subject. These values were then subtracted from the measured values resulting in age-corrected measurements for each feature.

#### Statistical analyses

Three binary logistic ridge regressions were fitted using (**a**) clinical indices alone (**b**) MRI features alone (**c**) and both clinical and MRI parameters. Clinical features included age at disease onset, site of disease onset (bulbar/spinal), diagnostic delay (time interval from symptom onset to diagnosis), and ALSFRS-r at the time of the scan. Table 2. MRI features corrected for age-related variability consisted of the average cortical thickness of the precentral gyrus, the average FA, RD, MD and AD of the superior corona radiata, inferior corona radiata, anterior and posterior limbs of the internal capsule, cerebral peduncles and the genu, body and splenium of the corpus callosum. Figure 3. The outcome variable was the probability of surviving less than 18 months. The statistical software R [34] and the package ‘glmnet’ (α = 0) [35] was utilised to carry out the logistic ridge regression. The tuning parameter λ was selected based on ten-folded cross-validation which was repeated 100 times. The model with the smallest misclassification error averaged over the 100 estimations was selected. Subsequently, the ridge regression algorithm was used to estimate the probability of each participant in the validation sample to belong to the group surviving less than 18 months after the brain scan.

**Table 2:** List of clinical and MRI features

Clinical indices	MRI features
• Age at disease onset	Cortical thickness
• Site of disease onset	• Precentral gyri
• Diagnostic delay	• Paracentral gyri
• Disease severity ALSFRS-r	White matter • Superior corona radiata
	• Inferior corona radiata
	• Anterior limbs of the internal capsule
	• Posterior limbs of the internal capsule
	• Cerebral peduncles
	• Genu of the corpus callosum
	• Body of the corpus callosum
	• Splenium of the corpus callosum

## Results

### Group comparisons

**Fig. 5:**
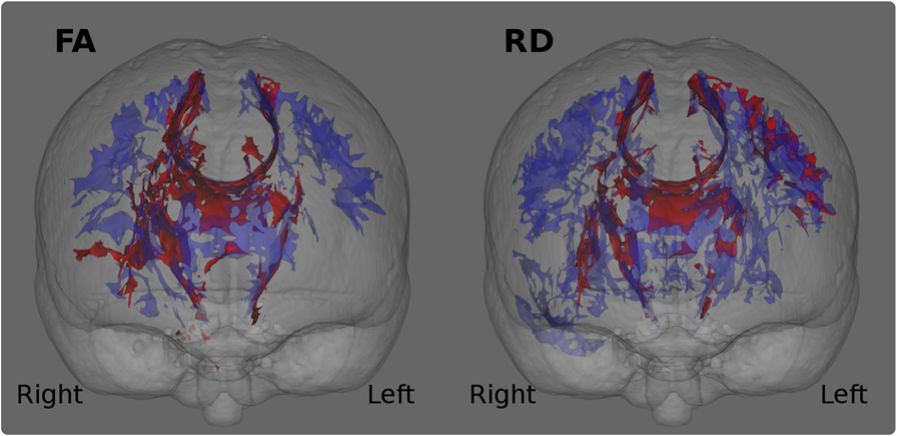
Patterns of white matter pathology in short and long survivors. *Blue* colour indicates *white* matter degeneration in ‘short survivals’ (<18 months) in comparison to controls, and *red* colour indicates *white* matter degeneration in ‘long survivors’ (>18 months) in comparison to controls at *p* < 0.05 (FWE) correcting for age

**Fig. 6:**
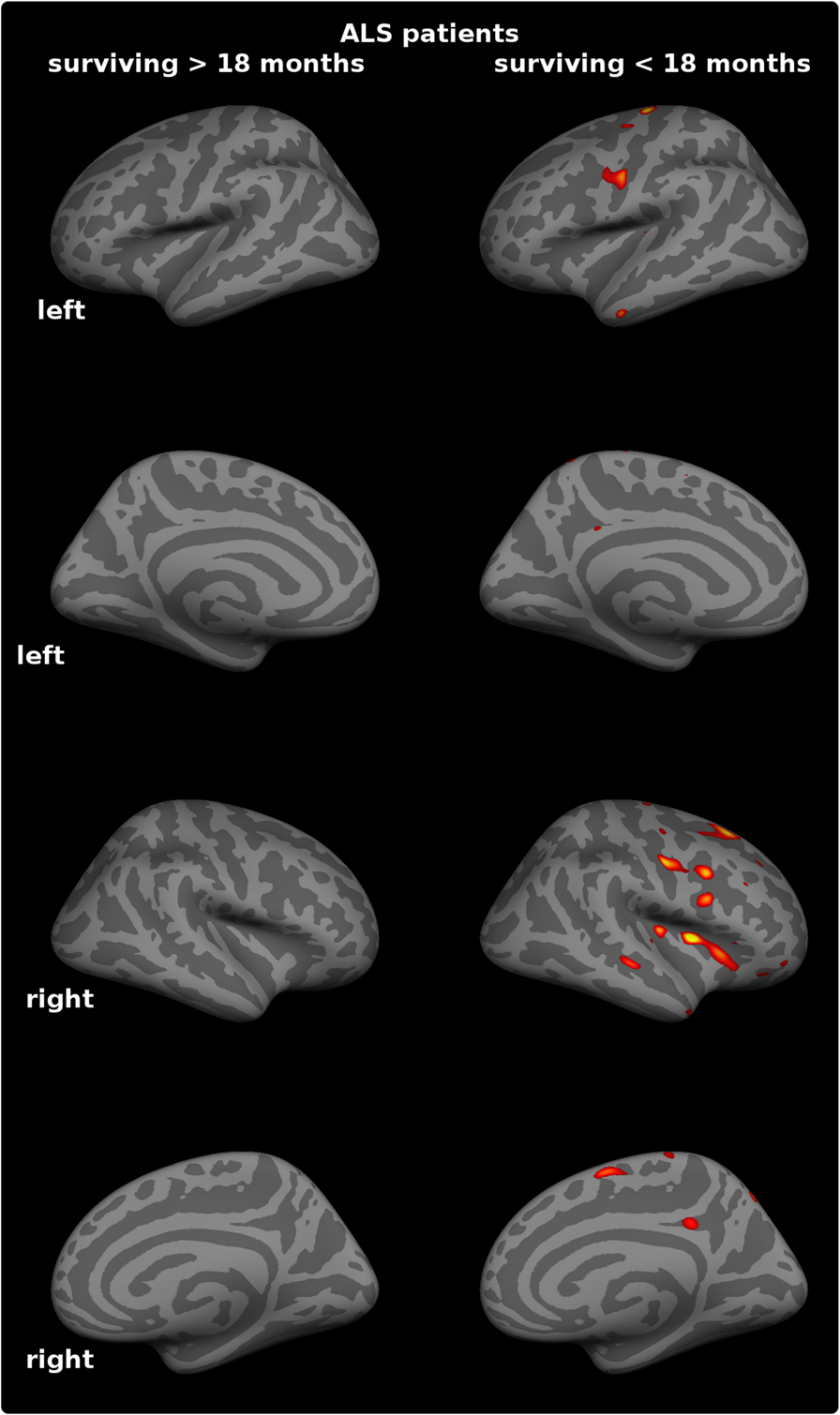
Patterns of cortical pathology in short and long survivors. Extensive, multifocal *grey* matter pathology is identified in ‘short survivals’ (<18 months) compared to controls at *p* < 0.05 (FDR) which is not captured at this statistical threshold in long-survivors. (>18 months)

The comparison of ALS patients in the training sample and controls highlighted ALS-specific patterns of neurodegeneration, i.e. cortical thinning of the precentral and paracentral gyri (Fig. 4) and white matter degeneration of all segments of the corticospinal tract and corpus callosum. (Figure 2). The direct comparison of patients surviving more than 18 months and those surviving less than 18 months, did not reach statistical significance with family-wise error corrections. However, patients with shorter survival have demonstrated significantly more widespread cortical and white matter changes in comparison to controls than those surviving longer Figs. 5 and 6.

**Fig. 7:**
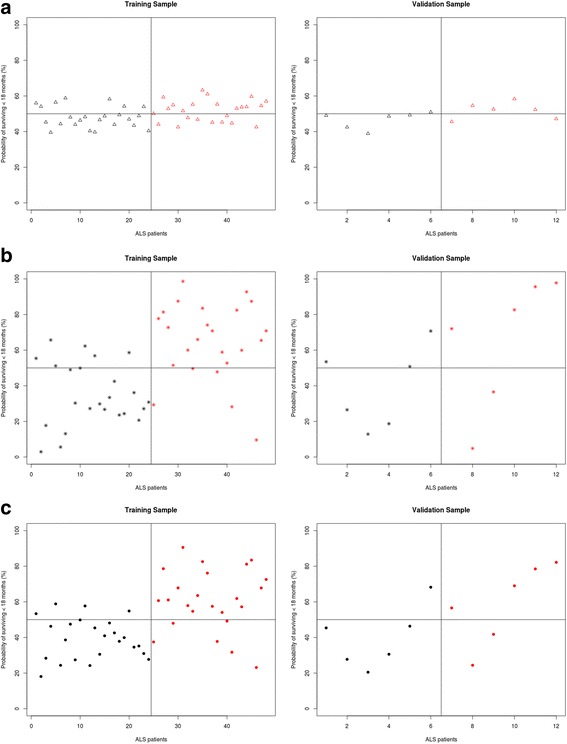
The predicted probability of surviving less than 18 months based on clinical variables (**a**) MRI variables (**b**) Clinical and MRI variables (**c**). *Red*: patients who have survived less than 18 months, *Black*: patients who have survived more than 18 months after their MRI scans

Figure 7 shows the probability of each patient to survive less than 18 months based on (**a**) clinical parameters, (**b**) MRI measures, and (**c**) both clinical and MRI indices. Using a cut-off score of 50%, the accuracy, sensitivity and specificity of prognostic classification in presented in Table 3. The coefficient estimates for each regression are presented in Additional file 1: Table S1.

**Table 3:** Classification outcomes using a 50% cut-off probability

A, Classification based on clinical characteristics alone				
Training sample				
Predicted class	Survival <18 months	Survival >18 months	Sensitivity	62.50%
True Class Survival <18 months	15	7	Specificity	70.84%
Survival >18 months	9	17	Accuracy	66.67%
Validation sample				
Predicted class	Survival <18 months	Survival >18 months	Sensitivity	66.67%
True Class Survival <18 months	4	1	Specificity	83.34%
Survival >18 months	2	5	Accuracy	75.00%
B, Classification based on MRI measures alone				
Training sample				
Predicted class	Survival <18 months	Survival >18 months	Sensitivity	79.16%
True Class Survival <18 months	19	6	Specificity	75.00%
Survival >18 months	5	18	Accuracy	77.08%
Validation sample				
Predicted class	Survival <18 months	Survival >18 months	Sensitivity	66.70%
True Class Survival <18 months	4	3	Specificity	50.00%
Survival >18 months	2	3	Accuracy	58.33%
C, Classification based on clinical and MRI measures				
Training sample				
Predicted class	Survival <18 months	Survival >18 months	Sensitivity	75.00%
True Class Survival <18 months	18	4	Specificity	83.34%
Survival >18 months	6	20	Accuracy	79.17%
Validation sample				
Predicted class	Survival <18 months	Survival >18 months	Sensitivity	66.67%
True ClassSurvival <18 months	4	1	Specificity	83.34%
Survival >18 months	2	5	Accuracy	75.00%

Analysis of misclassification revealed that among the clinical variables the total ALSFRS-R showed a significant difference between correctly classified and misclassified patients. When predicting a survival of less than 18 months, false negative patients had a higher ALSFRS-R in comparison to false positives who had lower ALSFRS-R scores (average ALSFRS-R for True Positive =30.93 (SD = 6.39), False Negative =40.11 (SD = 2.03), *p* < .01). Additional file 1: Table S2-13.

## Discussion

The presented study explores the role of MRI measures as prognostic biomarkers in ALS. While diagnostic and monitoring biomarkers have been extensively investigated in ALS, there is a scarcity of prognostic studies. One of the key finding of the study is the more widespread white and grey matter degeneration in ‘short-survivors’ compared to ‘long-survivors’. Figures 5 and 6. Based on these results, we aimed to systematically evaluate the value of structural MRI measures of disease-defining brain regions and clinical indices in predicting the probability of 18-month survival.

Based on the combination of structural brain measures and clinical characteristics, mortality within 18-months was predicted with relatively high accuracy; 79.17%. Moreover, 83.3% of patients were correctly identified as surviving for longer than 18 months following their brain scan, and 75% of the sample was correctly identified as surviving less than 18-months. Applying the regression algorithm to an independent validation sample further supports the validity of these findings. Despite the relatively small sample size of the validation cohort, the algorithm reached 75% accuracy. 83.34% of patients were correctly identified as surviving more than 18-months and 66.67% of patients were correctly identified as surviving less than 18 months. Based on MRI measures alone, the accuracy and sensitivity of the classification was similar, but the patients surviving more than 18 months were less likely to be identified correctly.

Using clinical and demographic measures alone, without MRI indices, prediction accuracy was considerably lower (66.67%). Similarly, the sensitivity and specificity profile of these predictions were inferior to the ones also incorporating MRI measures. These findings underscore the benefit of MRI measures of ALS-associated brain regions in predicting 18-months survival. (Table 3.)

Evaluating misclassified patients based on clinical features alone, the group incorrectly classified surviving less than 18 months was significantly less physically impaired. They had a higher score of the ALSFRS-R. In contrast, patients misclassified as surviving longer than 18 months, had significantly longer disease duration. Adding MRI measure, there was no difference found between misclassified patients, again emphasizing the benefit of this additional information.

Previous studies have linked MRI measures to survival. Two-year survival was predicted using motor cortex spectroscopy with a sensitivity of 67% and a specificity of 83% [21] and corticospinal tract diffusivity changes were utilised to predict three-year survival with a specificity of 61.5% and accuracy of 71.0%. [22] In contrast to previous studies, we present a multi-modal approach, assessing cortical thickness alterations in addition to the four most commonly used indices of white matter degeneration. Additionally, we test the generalisability of our classification method in an independent validation sample.

Accurate prognostic markers have a role in clinical management as well as in clinical trials. In the absence of effective disease-modifying therapies, the optimal timing of supportive measures [36], end-of-life decisions [37], palliative interventions [38] is particularly important in ALS. While ALS patients are eager to participate in clinical trials in all stages of the disease, it may be desirable to enrol relatively homogenous patient cohorts soon after their diagnosis, when limited neurodegenerative change has taken place. [2, 5] It is frequently the case however that heterogeneous patient cohorts are enrolled in clinical trials, encompassing diverse phenotypes in order to rapidly meet the targeted sample size [39]. In the era of precision medicine, therapeutic strategies and clinical trial designs should be tailored to specific phenotypes [40, 41] and disease stages [39]. For example, stem cells therapy is regarded to be less successful in bulbar onset ALS patients, and patients with advanced disease [42]. Clinical trials of specific phenotypes or homogenous cohorts may have other advantages, such as the inclusion of patients who are likely to progress at a relatively uniform rate. It has been proposed that the inclusion of patients with rapid progression rates may shorten clinical trials [43].

Using objective, validated and observer-independent prognostic markers, such as MRI measures, may be helpful for patient stratification into clinical trials. It is also important that study end-points, such as survival are independent from demographic factors. [41].

### Limitations and future directions

Our study outlines a prediction method based on single-time point MRI data, which is a snapshot of in vivo pathology at specific moment in the patient’s disease trajectory.

Survival prediction may be more accurate if multiple time-points are included and longitudinal change over time is considered. [2] Moreover, the inclusion of other disease-specific anatomical regions, such as basal ganglia [44, 45], spinal cord [46, 47], cerebellar [48] or electrophysiological measures [49] may improve prognostic categorisation further. As only patients scanned at least 18 months ago were included, the sample size of the study is relatively limited and 20% of the patients were randomly allocated to the validation sample to demonstrate the generalizability of the methods. The present pilot study outlines a proposed prognostic algorithm which should ideally be replicated in larger cohorts or data pooled from multiple centres. Other future directions include assessment of two-year survival, or other clinical milestones, such as introduction of non-invasive ventilation, walking aids, feeding tubes etc. In this study, the cognitive and behavioural profile of the patients were not considered, despite evidence that executive dysfunction is associated with shorter survival [7] and compliance with assistive devices. [36] We also acknowledge that, with the current MR technology, the additional prognostic value of MRI indices is limited, and may not be substantial enough to be incorporated in clinical trial designs.

## Conclusions

The combination of objective MRI measures and key clinical indices enable the accurate prediction of 18-month survival in ALS. Accurate, objective and validated prognostic markers are urgently required in ALS, and have implications both for clinical trial designs and individualised patient care.

## Additional file

Additional file 1: Table S1. Coefficients estimates of each logistic ridge regression.**Table S2.** Clinical features. Demographic and clinical data of correctly and misclassified patients surviving less than 18 months of the training sample using a cut-off of 50% probability. **Table S3.** Clinical features. Demographic and clinical data of correctly and misclassified patients surviving >18 months of the training sample using a cut-off of 50% probability. **Table S4.** Clinical features. Demographic and clinical data of correctly and misclassified patients surviving <18 months of the validation sample using a cut-off of 50% probability. **Table S5.** Clinical features. Demographic and clinical data of correctly and misclassified patients surviving >18 months of the validation sample using a cut-off of 50% probability. **Table S6.** MRI features. Demographic and clinical data of correctly and misclassified patients surviving <18 months of the training sample using a cut-off of 50% probability. **Table S7.** MRI features. Demographic and clinical data of correctly and misclassified patients surviving >18 months of the training sample using a cut-off of 50% probability. **Table S8.** MRI features. Demographic and clinical data of correctly and misclassified patients surviving <18 months of the validation sample using a cut-off of 50% probability. **Table S9.** MRI features. Demographic and clinical data of correctly and misclassified patients surviving >18 months of the validation sample using a cut-off of 50% probability. **Table S10.** Clinical and MRI features. Demographic and clinical data of correctly and misclassified patients surviving <18 months of the training sample using a cut-off of 50% probability. **Table S11.** Clinical and MRI features. Demographic and clinical data of correctly and misclassified patients surviving >18 months of the training sample using a cut-off of 50% probability. **Table S12.** Clinical and MRI features. Demographic and clinical data of correctly and misclassified patients surviving <18 months of the validation sample using a cut-off of 50% probability. **Table S13.** Clinical and MRI features. Demographic and clinical data of correctly and misclassified patients surviving >18 months of the validation sample using a cut-off of 50% probability. (DOCX 39 kb)

## Abbreviations

AD: Axial diffusivity
ALS: Amyotrophic lateral sclerosis
ALSFRS-R: The revised ALS functional rating scale
CT: Cortical thickness
DTI: Diffusion tensor imaging
FA: Fractional anisotropy
FDR: False discovery rate
FWR: Family wise error
HC: Healthy control
MD: Mean diffusivity
MRI: Magnetic resonance imaging
RD: Radial diffusivity
WM: White matter
