# Supplementary material for: Survival prediction in Amyotrophic lateral sclerosis based on MRI measures and clinical characteristics
Source: BMC Neurol. 2017 Apr 17;17:73. doi: 10.1186/s12883-017-0854-x (PMC5393027; doi:10.1186/s12883-017-0854-x)
Supplement: Additional file 1: Table S1. — Coefficients estimates of each logistic ridge regression.Table S2. Clinical features. Demographic and clinical data of correctly and misclassified patients surviving less than 18 months of the training sample using a cut-off of 50% probability. Table S3. Clinical features. Demographic and clinical data of correctly and misclassified patients surviving >18 months of the training sample using a cut-off of 50% probability. Table S4. Clinical features. Demographic and clinical data of correctly and misclassified patients surviving <18 months of the validation sample using a cut-off of 50% probability. Table S5. Clinical features. Demographic and clinical data of correctly and misclassified patients surviving >18 months of the validation sample using a cut-off of 50% probability. Table S6. MRI features. Demographic and clinical data of correctly and misclassified patients surviving <18 months of the training sample using a cut-off of 50% probability. Table S7. MRI features. Demographic and clinical data of correctly and misclassified patients surviving >18 months of the training sample using a cut-off of 50% probability. Table S8. MRI features. Demographic and clinical data of correctly and misclassified patients surviving <18 months of the validation sample using a cut-off of 50% probability. Table S9. MRI features. Demographic and clinical data of correctly and misclassified patients surviving >18 months of the validation sample using a cut-off of 50% probability. Table S10. Clinical and MRI features. Demographic and clinical data of correctly and misclassified patients surviving <18 months of the training sample using a cut-off of 50% probability. Table S11. Clinical and MRI features. Demographic and clinical data of correctly and misclassified patients surviving >18 months of the training sample using a cut-off of 50% probability. Table S12. Clinical and MRI features. Demographic and clinical data of correctly and misclassified patients surviving <18 months of the va [file 12883_2017_854_MOESM1_ESM.docx]

*Supporting information – Supplementary Tables*

|  | **Coefficient of** | | |
| --- | --- | --- | --- |
| **Feature** | **Clinical characteristics** | **MRI measures** | **Clinical and MRI measures** |
| Intercept | 0.75 | 0.20 | 0.72 |
| Age at disease onset | 0.01 |  | 0.02 |
| Site of disease onset | 0.03 |  | 0.06 |
| Diagnostic delay | -0.1 |  | -0.12 |
| Disease severity | -0.04 |  | -0.05 |
| CT – precentral gyri |  | -4.17 | -2.13 |
| CT – paracentral gyri |  | -3.386 | -1.6 |
| FA – superior corona radiata |  | -23.311 | -5.57 |
| FA – inferior corona radiata |  | 22.62 | 8.56 |
| FA – anterior limbs of the internal capsule |  | -3.96 | 4.80 |
| FA – posterior limbs of the internal capsule |  | 3.50 | -0.37 |
| FA – Cerebral peduncles |  | 8.04 | 1.76 |
| FA – Genu of the corpus callosum |  | -8.38 | -3.09 |
| FA – Body of the corpus callosum |  | -1.9 | 2.22 |
| FA – Splenium of the corpus callosum |  | 31.08 | 11.04 |
| RD– superior corona radiata |  | 5179.47 | 2297.60 |
| RD– inferior corona radiata |  | -2893.67 | -1042.0 |
| RD– anterior limbs of the internal capsule |  | -12256.40 | -4818.52 |
| RD– posterior limbs of the internal capsule |  | 4157.76 | 1778.07 |
| RD– Cerebral peduncles |  | 155.15 | 471.67 |
| RD– Genu of the corpus callosum |  | 5053.78 | 1134.02 |
| RD– Body of the corpus callosum |  | -5844.95 | -2532.94 |
| RD– Splenium of the corpus callosum |  | -5940.47 | -2812.46 |
| MD– superior corona radiata |  | 2304.21 | 2065.45 |
| MD– inferior corona radiata |  | 10876.3 | 4606.65 |
| MD– anterior limbs of the internal capsule |  | -5889.58 | -3351.52 |
| MD– posterior limbs of the internal capsule |  | 4264.42 | 2108.94 |
| MD– Cerebral peduncles |  | 2993.10 | 1409.014 |
| MD– Genu of the corpus callosum |  | -313.58 | -267.39 |
| MD– Body of the corpus callosum |  | -8191.43 | -2964.61 |
| MD– Splenium of the corpus callosum |  | 4883.16 | 1693.45 |
| AD– superior corona radiata |  | -4119.69 | 1267.91 |
| AD– inferior corona radiata |  | 18845.37 | 7960.91 |
| AD– anterior limbs of the internal capsule |  | 3106.76 | -649.61 |
| AD– posterior limbs of the internal capsule |  | 599.31 | 757.1 |
| AD– Cerebral peduncles |  | 3073.281 | 1117.36 |
| AD– Genu of the corpus callosum |  | -7106.65 | -1941.97 |
| AD– Body of the corpus callosum |  | -5984.16 | -1209.31 |
| AD– Splenium of the corpus callosum |  | 7342.6 | 2887.57 |

**Table S-1. Coefficients estimates of each logistic ridge regression.**

**Characteristics of misclassified patients**

| **Survival < 18 months** | | | |
| --- | --- | --- | --- |
|  | **True**  **Positive** | **False**  **Negative** | **p-value** |
| N | 15 | 9 |  |
| Gender (male/ female) | 11/ 4 | 6/ 3 | P = 1 |
| Age, years (means, SD) | 64.58 (9.64) | 60.85 (3.38) | P = .18 |
| Handedness (right/left) | 14/1 | 9/ 0 | P = 1 |
| Site of onset (non-spinal/spinal) | 3/ 12 | 5/ 4 | P = .18 |
| Diagnostic delay, years (mean, SD) | 1.14 (0.8) | 1.29 (0.88) | P = .68 |
| Disease duration from symptom onset until scan, years (mean, SD) | 2.18 (1.15) | 2.16 (0.78) | P = .97 |
| ALSFRS-r (mean, SD) | 30.93 (6.39) | 40.11 (2.03) | P < .01 |
| Survival from scan, years (mean, SD) | 0.86 (0.22) | 1.06 (0.44) | P = .23 |

**Table S-2 Clinical features.** Demographic and clinical data of correctly and misclassified patients surviving less than 18 months of the training sample using a cut-off of 50% probability.

| **Survival > 18 months** | | | |
| --- | --- | --- | --- |
|  | **True**  **Negative** | **False**  **Positive** | **p-value** |
| N | 17 | 7 |  |
| Gender (male/ female) | 10/ 7 | 3/ 4 | P = .79 |
| Age, years (means, SD) | 67.17 (11.73) | 59.52 (9.78) | P = .16 |
| Handedness, (right/left) | 14/ 3 | 6/ 1 | P = 1 |
| Site of onset (non-spinal/spinal) | 8/ 9 | 2/ 5 | P = .70 |
| Diagnostic delay, years (mean, SD) | 1.1 (0.85) | 0.93 (0.45) | P = .52 |
| Disease duration from symptom onset until scan, years (mean, SD) | 2.37 (1.21) | 2.2 (1.72) | P = .83 |
| ALSFRS-r (mean, SD) | 40.06 (2.79) | 30.86 (7.56) | P < .05 |
| Survival from scan, years (mean, SD) | 2.01 (0.58) | 2.87 (1.79) | P = .26 |

**Table S-3 Clinical features**. Demographic and clinical data of correctly and misclassified patients surviving > 18 months of the training sample using a cut-off of 50% probability.

| **Survival < 18 months** | | | |
| --- | --- | --- | --- |
|  | **True**  **Positive** | **False**  **Negative** | **p-value** |
| N | 4 | 2 |  |
| Gender (male/ female) | 3/ 1 | 0/ 2 | P = .39 |
| Age, years (means, SD) | 60.81 (6.38) | 70.25 (9.02) | P =.35 |
| Handedness (right/left) | 4/ 0 | 1/ 1 | P = .69 |
| Site of onset (non-spinal/spinal) | 1/ 3 | 2/ 0 | P = .39 |
| Diagnostic delay, years (mean, SD) | 1.54 (1.32) | 0.75 (0.11) | P = .32 |
| Disease duration from symptom onset until scan, years (mean, SD) | 2.34 (1.67) | 1.14 (0.15) | P = .25 |
| ALSFRS-r (mean, SD) | 30.75 (7.68) | 43 (1.41) | P = .06 |
| Survival from scan, years (mean, SD) | 1.01 (0.28) | 0.74 (0.08) | P = .14 |

**Table S-4 Clinical features.** Demographic and clinical data of correctly and misclassified patients surviving < 18 months of the validation sample using a cut-off of 50% probability.

| **Survival > 18 months** | | | |
| --- | --- | --- | --- |
|  | **True**  **Negative** | **False**  **Positive** | **p-value** |
| N | 5 | 1 |  |
| Gender (male/ female) | 2/3 | 0/1 | - |
| Age, years (means, SD) | 52.64 (7.21) | 67.38 (NA) | - |
| Handedness (right/left) | 4/ 1 | 1/ 0 | - |
| Site of onset (non-spinal/spinal) | 2/ 3 | 0/ 1 | - |
| Diagnostic delay, years (mean, SD) | 0.91 (0.32) | 0.75 (NA) | - |
| Disease duration from symptom onset until scan, years (mean, SD) | 2.01 (0.41) | 1.05 (NA) | - |
| ALSFRS-r (mean, SD) | 38.8 (4.55) | 41 (NA) | - |
| Survival from scan, years (mean, SD) | 2.83 (1.36) | 1.56 (NA) | - |

**Table S-5 Clinical features.** Demographic and clinical data of correctly and misclassified patients surviving > 18 months of the validation sample using a cut-off of 50% probability.

| **Survival < 18 months** | | | |
| --- | --- | --- | --- |
|  | **True**  **Positive** | **False**  **Negative** | **p-value** |
| N | 19 | 5 |  |
| Gender (male/ female) | 14/ 5 | 3/2 | P =.96 |
| Age, years (means, SD) | 62.94 (8.26) | 64.12 (7.7) | P = .77 |
| Handedness (right/left) | 18/ 1 | 5/0 | P = 1 |
| Site of onset (non-spinal/spinal) | 5/14 | 3/ 2 | P = .38 |
| Diagnostic delay, years (mean, SD) | 1.09 (0.71) | 1.61 (1.13) | p = .37 |
| Disease duration from symptom onset until scan, years (mean, SD) | 2.14 (1.01) | 2.28 (1.1) | P = .80 |
| ALSFRS-r (mean, SD) | 34.05 (7.26) | 35.6 (5.46) | P = .61 |
| Survival from scan, years (mean, SD) | 0.91 (0.34) | 1.05 (0.23) | P = .30 |

**Table S-6 MRI features**. Demographic and clinical data of correctly and misclassified patients surviving < 18 months of the training sample using a cut-off of 50% probability.

| **Survival > 18 months** | | | |
| --- | --- | --- | --- |
|  | **True**  **Negative** | **False**  **Positive** | **p-value** |
| N | 18 | 6 |  |
| Gender (male/ female) | 8/ 10 | 5/ 1 | P = .24 |
| Age, years (means, SD) | 62.31 (10) | 60.09 (13.61) | P = .72 |
| Handedness, (right/left) | 14/ 4 | 6/ 0 | P = .53 |
| Site of onset (non-spinal/spinal) | 6/ 12 | 4/ 2 | P = .34 |
| Diagnostic delay, years (mean, SD) | 1.08 (0.79) | 0.96 (0.64) | P = .73 |
| Disease duration from symptom onset until scan, years (mean, SD) | 2.42 (1.38) | 2.03 (1.27) | P = .54 |
| ALSFRS-r (mean, SD) | 37.5 (6.05) | 37 (7.27) | P =.88 |
| Survival from scan, years (mean, SD) | 2 (0.57) | 3.07 (1.87) | P =.22 |

**Table S-7 MRI features**. Demographic and clinical data of correctly and misclassified patients surviving > 18 months of the training sample using a cut-off of 50% probability.

| **Survival < 18 months** | | | |
| --- | --- | --- | --- |
|  | **True**  **Positive** | **False**  **Negative** | **p-value** |
| N | 4 | 2 |  |
| Gender (male/ female) | 2/2 | 1/1 |  |
| Age, years (means, SD) | 66.4 (8.42) | 59.08 (6.18) | P = .31 |
| Handedness (right/left) | 3/1 | 2/0 | P = 1 |
| Site of onset (non-spinal/spinal) | 3/1 | 0/2 | P = .38 |
| Diagnostic delay, years (mean, SD) | 1.5 (1.34) | 0.84 (0.33) | P = .41 |
| Disease duration from symptom onset until scan, years (mean, SD) | 1.84 (1.44) | 2.14 (1.99) | P = 0.87 |
| ALSFRS-r (mean, SD) | 35.25 (9.46) | 33 (8.49) | P = .79 |
| Survival from scan, years (mean, SD) | 0.79 (0.08) | 1.19 (0.35) | P = .34 |

**Table S-8 MRI features**. Demographic and clinical data of correctly and misclassified patients surviving < 18 months of the validation sample using a cut-off of 50% probability.

| **Survival > 18 months** | | | |
| --- | --- | --- | --- |
|  | **True**  **Negative** | **False**  **Positive** | **p-value** |
| N | 3 | 3 |  |
| Gender (male/ female) | 2/ 1 | 0/ 3 | P = .39 |
| Age, years (means, SD) | 50.47 (5.83) | 59.72 (9.81) | P = .25 |
| Handedness (right/left) | 2/ 1 | 3/ 0 | P = 1 |
| Site of onset (non-spinal/spinal) | 1/ 2 | 1/ 2 | P = 1 |
| Diagnostic delay, years (mean, SD) | 0.88 (0.26) | 0.89 (0.39) | P =.97 |
| Disease duration from symptom onset until scan, years (mean, SD) | 1.97 (0.57) | 1.74 (0.6) | P =.66 |
| ALSFRS-r (mean, SD) | 41.33 (1.15) | 37 (5.29) | P = .29 |
| Survival from scan, years (mean, SD) | 2.96 (1.72) | 2.28 (1.03) | P = .59 |

**Table S-9 MRI features**. Demographic and clinical data of correctly and misclassified patients surviving > 18 months of the validation sample using a cut-off of 50% probability.

**Clinical and MRI features**

| **Survival < 18 months** | | | |
| --- | --- | --- | --- |
|  | **True**  **Positive** | **False**  **Negative** | **p-value** |
| N | 18 | 6 |  |
| Gender (male/ female) | 13/ 5 | 4/ 2 | P = 1 |
| Age, years (means, SD) | 63.38 (8.69) | 62.59 (6.09) | P = .81 |
| Handedness (right/left) | 17/ 1 | 6/ 0 | P =1 |
| Site of onset (non-spinal/spinal) | 6/ 12 | 2/ 4 | P = 1 |
| Diagnostic delay, years (mean, SD) | 1.03 (0.69) | 1.71 (1.01) | p = .17 |
| Disease duration from symptom onset until scan, years (mean, SD) | 1.99 (0.79) | 2.71 (1.43) | P = .29 |
| ALSFRS-r (mean, SD) | 34 (7.21) | 35.5 (6.06) | P = .63 |
| Survival from scan, years (mean, SD) | 0.97 (0.3) | 0.84 (0.4) | P = .50 |

**Table S-10 Clinical and MRI features**. Demographic and clinical data of correctly and misclassified patients surviving < 18 months of the training sample using a cut-off of 50% probability.

| **Survival > 18 months** | | | |
| --- | --- | --- | --- |
|  | **True**  **Negative** | **False**  **Positive** | **p-value** |
| N | 20 | 4 |  |
| Gender (male/ female) | 10/ 10 | 3/ 1 | P = .71 |
| Age, years (means, SD) | 61.82 (10.7) | 61.42 (13.72) | P = .96 |
| Handedness, (right/left) | 16/ 4 | 4/ 0 | P = .81 |
| Site of onset (non-spinal/spinal) | 8/ 12 | 2/ 2 | P = 1 |
| Diagnostic delay, years (mean, SD) | 1.11 (0.77) | 0.73 (0.58) | P = .30 |
| Disease duration from symptom onset until scan, years (mean, SD) | 2.46 (1.41) | 1.64 (0.65) | P = .10 |
| ALSFRS-r (mean, SD) | 37.95 (5.9) | 34.5 (7.9) | P = .46 |
| Survival from scan, years (mean, SD) | 1.98 (0.54) | 3.66 (2.11) | P = .21 |

**Table S-11 Clinical and MRI features**. Demographic and clinical data of correctly and misclassified patients surviving > 18 months of the training sample using a cut-off of 50% probability.

| **Survival < 18 months** | | | |
| --- | --- | --- | --- |
|  | **True**  **Positive** | **False**  **Negative** | **p-value** |
| N | 4 | 2 |  |
| Gender (male/ female) | 2/2 | 1/ 1 | P = 1 |
| Age, years (means, SD) | 66.4 (8.42) | 59.08 (6.18) | P = .32 |
| Handedness (right/left) | 3/ 1 | 2/ 0 | P = 1 |
| Site of onset (non-spinal/spinal) | 3/ 1 | 0 / 2 | P = .39 |
| Disease duration from onset until diagnosis, years (mean, SD) | 1.5 (1.34) | 0.84 (0.33) | P = .40 |
| Disease duration from symptom onset until scan, years (mean, SD) | 1.84 (1.44) | 2.14 (1.99) | P = .87 |
| ALSFRS-r (mean, SD) | 35.25 (9.46) | 33 (8.49) | P = .79 |
| Survival from scan, years (mean, SD) | 0.79 (0.08) | 1.19 (0.35) | P = .35 |

**Table S-12 Clinical and MRI features**. Demographic and clinical data of correctly and misclassified patients surviving < 18 months of the validation sample using a cut-off of 50% probability.

| **Survival < 18 months** | | | |
| --- | --- | --- | --- |
|  | **True**  **Negative** | **False**  **Positive** | **p-value** |
| N | 5 | 1 |  |
| Gender (male/ female) | 2/ 3 | 0/ 1 | - |
| Age, years (means, SD) | 52.64 (7.21) | 67.38 (NA) | - |
| Handedness (right/left) | 4/ 1 | 1 / 0 | - |
| Site of onset (non-spinal/spinal) | 2/ 3 | 0/ 1 | - |
| Diagnostic delay, years (mean, SD) | 0.91 (0.32) | 0.75 (NA) | - |
| Disease duration from symptom onset until scan, years (mean, SD) | 2.01 (0.41) | 1.05 (NA) | - |
| ALSFRS-r (mean, SD) | 38.8 (4.55) | 41 (NA) | - |
| Survival from scan, years (mean, SD) | 2.83 (1.36) | 1.56 (NA) | - |

**Table S-13 Clinical and MRI features**. Demographic and clinical data of correctly and misclassified patients surviving > 18 months of the validation sample using a cut-off of 50% probability.
